# Persistent SARS-CoV-2 Infection in a Multiple Sclerosis Patient on Ocrelizumab: A Case Report

**DOI:** 10.21203/rs.3.rs-2768759/v1

**Published:** 2023-04-04

**Authors:** Raghava S. Ambadapoodi, Forest W. Arnold, Julia H. Chariker, Alex Glynn, William Lauer, Subathra Marimuthu, Eric C. Rouchka, Melissa L. Smith, Leslie A. Wolf

**Affiliations:** 1 Department of Neurology, University of Chicago Medical Center, Chicago, IL; 2 Division of Infectious Diseases, School of Medicine, University of Louisville, Louisville, KY; 3 Neuroscience Training Department, University of Louisville, Louisville, KY; 4 Department of Biochemistry and Molecular Genetics, University of Louisville, Louisville, KY

**Keywords:** SARS-CoV-2, multiple sclerosis, ocrelizumab, immunocompromised, persistent infection, long-read sequencing

## Abstract

A 44-year-old female patient with multiple sclerosis (MS) treated with ocrelizumab was hospitalized with SARS-CoV-2 pneumonia three times over the course of five months, eventually expiring. Viral sequencing of samples from her first and last admissions suggests a single persistent SARS-CoV-2 infection. We hypothesize that her immunocompromised state, due to MS treatment with an immunosuppressive monoclonal antibody, prevented her from achieving viral clearance.

## Introduction

Ocrelizumab is an anti-CD20 monoclonal antibody approved by the U.S. Food & Drug Administration (FDA) since 2017 for the management and treatment of primary progressive and relapsing multiple sclerosis (MS) in adults.[1] It causes B-cell depletion, by which means it limits cytokine regulation, antigen presentation, and autoantibody production, leading to improved clinical outcomes in multiple sclerosis (MS). Here, we describe the case of a patient on ocrelizumab for MS who suffered a persistent SARS-CoV-2 infection lasting more than three months, during which time the virus appears to have mutated *in vivo*. This phenomenon may have been caused by the patient’s B-cell-depleted state.

## Case Description

A 44-year-old woman with MS tested positive for SARS-CoV-2 infection at an outpatient testing facility, then presented two weeks later to the emergency department with fever, nausea, watery diarrhea, cough, and decreased oral intake. A nasopharyngeal swab tested negative for SARS-CoV-2 by polymerase chain reaction (PCR). She did not complain of shortness of breath or chest pain. Her medical history was notable for MS—treated with ocrelizumab 600 mg every six months with the most recent dose given four months prior to admission—and childhood asthma.

At presentation, her temperature was 99.9 °F, heart rate 118–121 beats/minute, respiratory rate 28–31 breaths/minute, and blood pressure 121/77 mmHg. Her oxygen saturation was 93% on room air. A physical exam revealed that she was alert and oriented, in no acute distress, with lungs clear to auscultation bilaterally and normal heart sounds without gallops or murmur.

**Figure 1.**
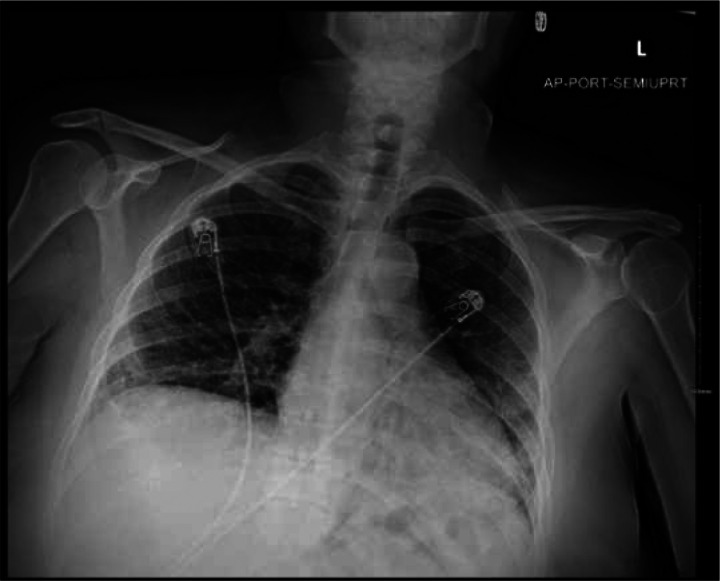
Portable chest X-ray on presentation to the Emergency Department (admission 1, day 1).

A complete blood count, arterial blood gas, and other laboratory values were obtained (Tables 1 and 2). Stool pathogen and *Clostridium difficile* assays were negative. A portable chest X-ray (CXR) was obtained (Figure 1). A single dose of vancomycin and cefepime was administered. Other in-patient medications included azithromycin, ceftriaxone, dexamethasone 6 mg daily, and baclofen.

**Figure 2.**
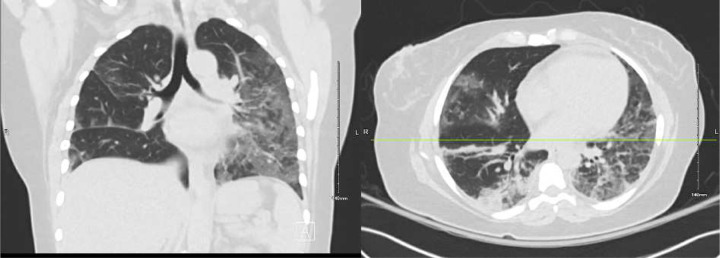
Chest computed tomography without contrast (admission 1, day 2), showing multifocal, mixed airspace and ground glass opacities extending towards the periphery of the lungs.

A chest computed tomography (CT) was obtained on hospital day 2 (Figure 2). Azithromycin and ceftriaxone were started. A bronchoalveolar lavage was performed on hospital day 4, yielding a sample positive for SARS-CoV-2 by PCR. CXRs worsened, antimicrobial coverage was broadened, and remdesivir and dexamethasone were started. On hospital day 5, she developed a pneumothorax, prompting transfer to the intensive care unit (ICU), where a chest tube was placed. She was discharged on hospital day 11 on home oxygen and two more days of dexamethasone.

**Figure 3.**
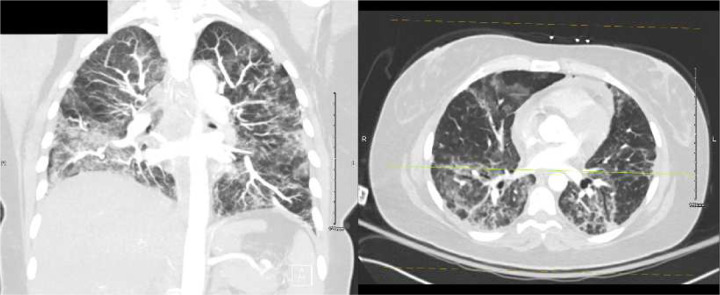
Outpatient computed tomography one day before second admissions, showing worsening multifocal pneumonia.

**Figure 4.**
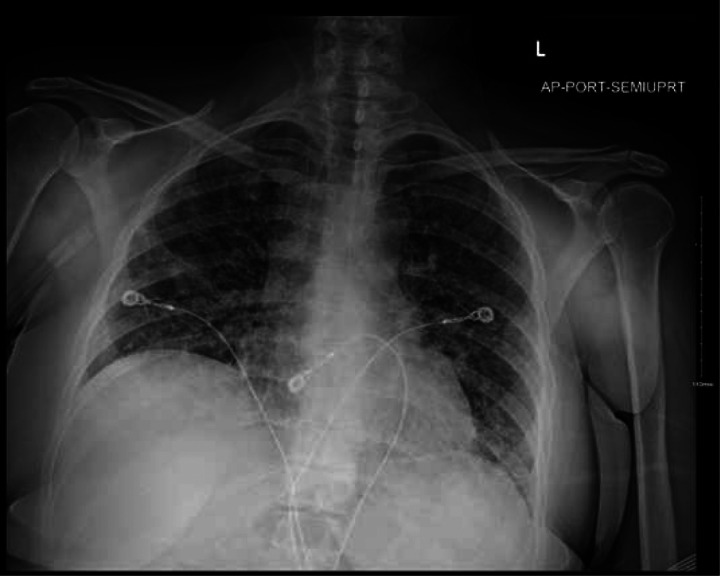
Portable chest X-ray on admission the patient’s second admission to the hospital.

Six days later, an outpatient CT showed worsening multifocal pneumonia (Figure 3), and the following day, the patient was readmitted to the hospital with fever. A portable CXR and chest pulmonary angiogram were consistent with worsening multifocal pneumonia (Figure 4). Her vital signs were abnormal, prompting ICU care, and she was started on methylprednisolone 30 mg BID. A sputum Gram’s stain showed Gram-positive cocci, suggestive of *Streptococcus pneumoniae*. Her condition improved, and she was ultimately discharged in stable condition on home oxygen 3–4 L by nasal cannula (NC), prednisone 50 mg daily with a planned taper, and outpatient therapy.

**Figure 5.**
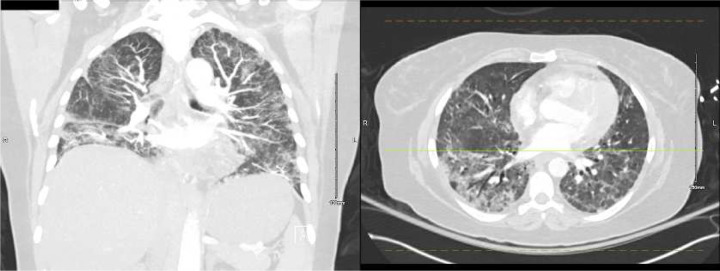
Outpatient chest computed tomography two days before the patient’s third admission to the hospital, showing ground glass and airspace opacities in the lower lungs differening in distribution from the first admission, with a few focal areas of honeycombing, most prominently in the left upper lobe.

About three months later, an outpatient chest CT was performed for suspected post-COVID-19 syndrome, showing ground glass and airspace opacities in the lower lungs, which differed in distribution from the CT performed during the first admission, with a few focal areas of honeycombing, most prominently in the left upper lobe (Figure 5). The new CT also showed increased measurement of the main pulmonary artery, concerning for pulmonary hypertension.

The patient presented to the emergency department two days later complaining of shortness of breath due to a panic attack. A nasopharyngeal swab tested negative for COVID-19 by PCR, and the patient was diagnosed with severe respiratory distress. A chest CT pulmonary angiogram was negative for pulmonary embolism but showed pulmonary fibrosis. Her oxygen saturation was initially low (83%) but improved on non-rebreather mask, followed by high-flow NC, and she was transferred to the ICU.

A nystatin suspension was given for an oral thrush present on admission. She did not receive her twice-yearly ocrelizumab infusion due to recent COVID-19 exposure and frequent hospitalizations. Her prednisone was increased to 47.5 mg daily. A bronchoscopy with bronchoalveolar lavage (BAL) and cryobiopsy was performed on hospital day 5. BAL was positive for COVID-19 (cycle threshold values: RNase P 24, N1 30.4, N3 28.8), and cryobiopsy showed pulmonary fibrosis; however, she developed severe intrapulmonary hemorrhage during the procedure, manifested by diffuse opacities on CXR. She was started on trimethoprim/sulfamethoxazole for Pneumocytis pneumonia prophylaxis.

The patient had fluctuating oxygen requirements; she was continued on 40 mg prednisone with a planned taper to 10 mg daily. On hospital day 9, she began a course of fluconazole for an esophageal thrush caused by prolonged steroid use.

She was discharged to a rehabilitation clinic with plans to continue fluconazole for another 17 days and taper her prednisone 5 mg per week. Three weeks later, the patient was discharged from the rehabilitation facility; she expired at another hospital due to cardiopulmonary arrest a few weeks thereafter.

### Sample analysis

The patient’s BAL samples from admissions 1 (NOV2020) and 3 (MAR2021), which were positive for SARS-CoV-2 by real-time PCR, were analyzed using a Mass ARRAY^®^ SARS-CoV-2 Variant Panel from Agena Bioscience^®^ at Louisville Metro Public Health Laboratory. This variant panel assay is designed to overcome sequencing-based technologies challenges with a high throughput, low-cost, and rapid assay for the detection of 36 unique genetic markers and differentiation of key SARS-CoV-2 variants. Samples from the patient’s first and third visits both had the same mutation, D614G; no other mutations were detected by Mass ARRAY^®^ (**Supplemental Table 1**).

**Figure 6.**
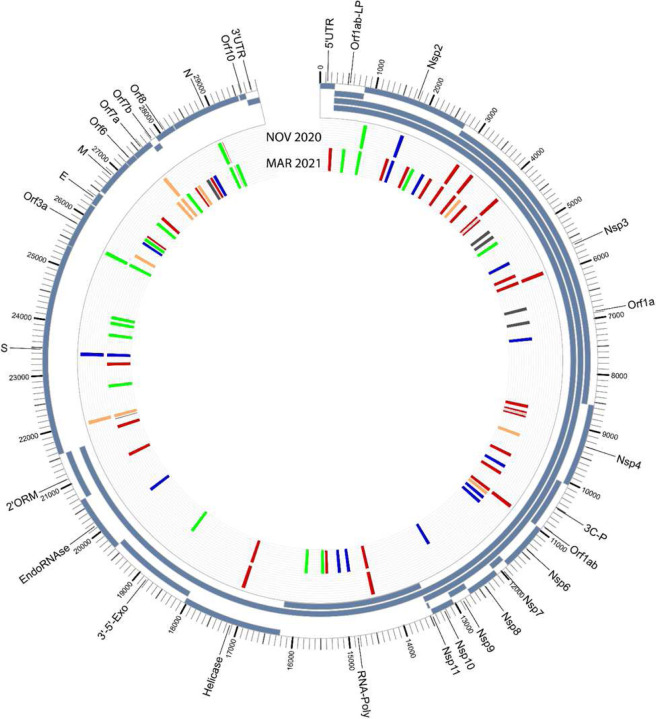
Circos plot showing the mutations for the NOV2020 sample (outer colored tick marks) and the MAR2021 sample (inner colored tick marks).

**Figure 7.**
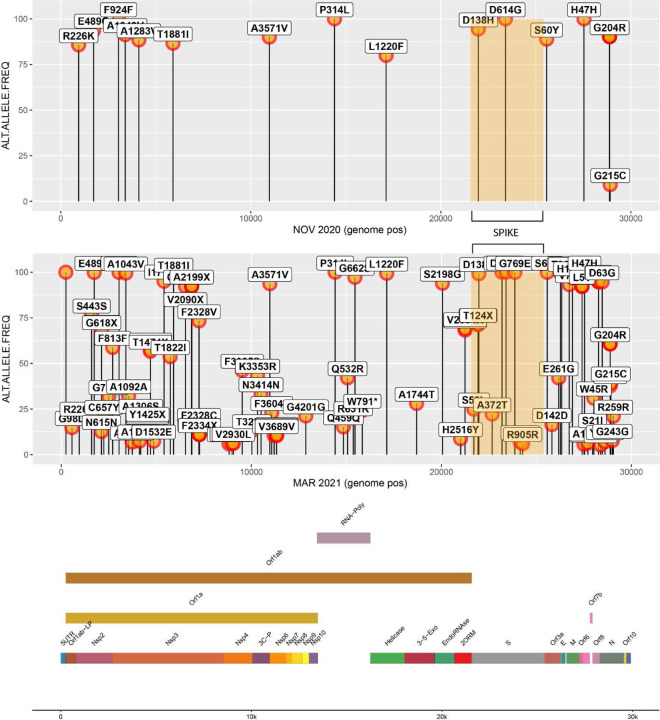
Lollipop graph of mutations detected within the NOV2020 sample (below) and MAR2021 sample (above).

The samples were also sequenced at the University of Louisville Sequencing Technology Center using PacBio long-read sequencing methodologies. The analysis yielded a Pangolin lineage assignment of B.1.1.186 with all seven of the mutations associated with the lineage for NOV2020 [2]; an additional seven mutations were also found in NOV2020. For MAR2021, a Pangolin lineage assignment of B.1.462 was given, initially suggestive of a separate lineage. However, upon further analysis, all of the mutations identified in NOV2020 were also found in MAR2021, with a large number of additional mutations also identified, yielding a total of 103 mutations (Figures 6–7, **Supplemental Table 2**).

## Discussion

On MASS Array^®^ analysis, BAL samples from the patient’s first and third visits both had the same mutation, D614G, suggestive of a persistent infection, rather than multiple infections. D614G was the only mutation detected in either sample by Mass ARRAY^®^. SARS-CoV-2 wild type virus is B.1 with D614G and no other spike protein changes. The spike protein mutation D614G is present in almost all emergent variants. Korber *et al*. were able to identify the SARS-CoV-2 spike protein D614G point mutation using the database of the extensive worldwide sequencing effort GISAID (Global Initiative for Sharing All Influenza Data).[9] This adenine to guanine nucleotide mutation (aspartic acid to glycine shift at the amino acid position 614 of a spike protein) at position 23,403 in the Wuhan reference strain, was the first mutation identified in early March 2020. Korber *et al*. also found that this point mutation is associated with high viral load in the upper respiratory tract in human patients, as well as higher infectivity and transmissibility. However, our patient’s COVID-19 test results—negative on nasopharyngeal swabs but positive on BAL—indicate that the infection was concentrated in the lower respiratory tract.

Long-read sequencing (PacBio) data analysis of the same BAL samples suggests that once this patient was infected with SARS-CoV-2, the virus was never cleared; rather, the patient was chronically infected, leading to long-term intra-host adaptation. The genetic variation seen in the MAR2021 sample was extensive, with a total of 103 mutations; however, carriage of the same mutational profile from the NOV2020 is strongly suggestive that this evolution occurred *in vivo* and was not the result of a subsequent, community-acquired infection. This is further supported by the larger SARS-CoV-2 variant community circulation patterns in Louisville, KY, in March 2021—dominated by the B.1.1.7 lineage, which did not demonstrate the same mutational profile observed in the B.1.2 lineage, which was dominant in November 2020. The extensive expansion of mutations in the MAR2021 sample is further supported by several examples of dramatic intra-host SARS-CoV-2 evolution in the context of immunosuppression or compromised immune system [3–6], such as was the case in this patient. In fact, it has been speculated that the dramatic genetic shifts seen in emergent, highly infectious SARS-CoV-2 lineages, such as Omicron, characterized by the acquisition of multiple concurrent mutations, were derived from an immunocompromised individual.[7, 8]

### Persistent SARS-CoV-2 infection

To our knowledge, this report describes the longest period of chronic SARS-CoV-2 infection in the literature for an MS a patient on ocrelizumab. Other cases of prolonged SARS-CoV-2 infection in immunocompromised patients, including those on anti-CD20 and other disease-modifying therapies, have been reported. A systematic review identified nine such cases ranging from 91 to >250 days.[10] Eight of the nine patients had B-cell immunodeficiency, and four were receiving B-cell-depleting therapies, but none were MS patients, and none were on ocrelizumab. New mutations emerging over time were detected in all nine patients. Time-measured phylogenetic reconstruction indicated that SARS-CoV-2 evolution was faster in these patients than in the general population.

Other cases in the literature include a patient with stage IV follicular lymphoma, whose therapies included rituximab (anti-CD20) and glofitamab (anti-CD20/anti-CD3 bispecific).[11] In this patient, infection persisted for over 200 days. Viral sequence analyses of samples from days 29, 79, and 150 showed the B.1.389 lineage with no indication of superinfection, suggesting a single chronic infection, much like our patient. Another similar feature was the identification of the D614G mutation in all three samples.

Chaudry *et al*. reported the case of a 62-year-old man with a history of non-Hodgkin’s lymphoma—treated with rituximab, an anti-CD20 monoclonal antibody similar to ocrelizumab—who persistently tested positive for SARS-CoV-2 by PCR for over 230 days.[12] Dulu *et al*. documented an even longer course of infection, with six positive and two negative PCR tests across 12 months.[13] This patient had diffuse large B-cell lymphoma, also treated with rituximab. He was hospitalized seven times over the course of infection. Dulu *et al*. suggest that due to B-cell dysfunction and treatment depletion of B-cells, lymphoma patients may shed persistently across extended periods. A similar phenomenon may have occurred in our patient due to her B-cell-depleting therapy.

Gibson *et al*. reported a case of SARS-CoV-2 infection in a patient on ocrelizumab lasting 69 days, with viral RNA shedding, signs, and symptoms persistent throughout.[14] In this case, the patient’s condition resolved after the administration of convalescent plasma and antiviral therapy. Lee *et al*. found that among 368 COVID-19 patients with hematologic malignancies, treatment with anti-CD20 therapies within one year of SARS-CoV-2 infection was associated with three times the odds of a positive test ≥ 30 days after initial positivity (OR 3.04 [95% CI 1.49–6.15]).[11]

The association between anti-CD20 therapy and prolonged SARS-CoV-2 infection is far from clear-cut, however. Novi *et al*. described the case of a patient treated with ocrelizumab whose COVID-19 symptoms abated a few days after hospital admission despite complete B-cell depletion.[15] Epstein *et al*. studied 3,758 adults retested within 90 days of an initial positive SARS-CoV-2 result and found that solid organ transplant recipients were at increased risk of delayed PCR clearance (aHR 0.64 [95% CI 0.42–0.97]), but this association was observed in neither severely nor moderately immunocompromised persons overall compared to immunocompetent persons (HR 0.98 [95% CI 0.84–1.15] and 0.86 [95% CI 0.70–1.05], respectively).[16]

### Infection, severity, and outcomes

A retrospective analysis found that among 30,478 persons with MS and an open prescription for diseasemodfying therapy (DMT), 344 were diagnosed with COVID-19, yielding an incidence of 1.13%.[17] Those on ocrelizumab or rituximab had significantly higher odds of developing COVID-19 than those on fumerates (OR 3.25 [95% CI 2.31–4.64]), anti-VLA-4 antibodies (OR 2.32 [95% CI 1.56–3.57]), or interferon (OR 4.65 [95% CI 3.23–6.82]). Smith *et al*. found no association between ocrelizumab and COVID-19; however, theirs was a single-center study with a smaller sample size (*n*=230).[18]

A study of 844 MS patients with COVID-19 found that anti-CD20 therapy (ocrelizumab or rituximab) was significantly associated with more than twice the odds of severe COVID-19 (OR 2.37 [95% CI 1.18–4.74], *P*=0.015).[20] In an analysis of 2,340 COVID-19 patients with MS, those treated with ocrelizumab (*n*=471) had significantly increased odds of hospitalization (OR 1.56 [95% CI 1.01–2.41]), but not ICU admission, artificial ventilation, or death, compared to those on dimethyl fumerate and natalizumab.[19]

Hughes *et al*. found similar rates of hospitalization, invasive ventilation, and mortality among patients who were (*n*=48) and were not (*n*=309) taking ocrelizumab and no association between duration of exposure to ocrelizumab and COVID-19 infection; among 307 cases of COVID-19 in the Roche/Genentech post-marketing safety data for ocrelizumab, less than one third were hospitalized (32.6%) and nearly half were asymptomatic, mild, or moderate (46.6%).[21] Bsteh *et al*. found that neither exposure to any DMT nor to any specific immunosuppressive DMT was significantly associated with COVID-19 severity (OR 1.6, *P*=0.667 and OR 1.9, *P*=0.426, respectively).[22] Czarnowszka *et al*. studied 396 patients on DMTs for MS, 20 (5.05%) of whom were on ocrelizumab, and found no significant difference in severity of SARS-CoV-2 infection according to the type of DMT used.[23] We cannot, therefore, be certain that our patient’s ocrelizumab treatment was responsible for the severity or outcomes of her illness.

### Immune response

There is some evidence to suggest that B-cell depletion associated with ocrelizumab can limit the immune response of MS patients to SARS-CoV-2 infection. A case-control study of 24 patients with MS and PCR-confirmed COVID-19 found that those on ocrelizumab (*n*=15) had significantly lower odds of forming antibodies compared to those on other DMTs (OR 0.045 [0.004–0.488], *P*=0.011).[24]

Similarly, Habek *et al*. found that B-cell-depleting therapy was an independent predictor of negative IgG for SARS-CoV-2 compared to healthy controls (Exp[B]=0.014 [95% CI 0.002–0.110], *P*<0.001), while other DMTs were not (Exp[B]=0.142 [95% CI 0.014–1.424], *P*=0.097).[25]

A single-center prospective study of 119 patients, 21 of whom were on anti-CD20 therapy, found that treatment with anti-CD20 therapy was associated with decreased odds of positive serology (OR 0.07 [95% CI 0.01–0.69; *P*=0.02]) and decreased anti-S IgG titer (estimate: −1.06 [95% CI −1.79 to −0.34], *P*=0.004).[26] A lesser or absent immune response may explain the lack of viral clearance in our case; unfortunately, antibody testing was not performed.

### Conclusions

Despite a lack of consensus in the medical literature as to the impact of ocrelizumab and other DMTs on patients with COVID-19, it is likely that our patient’s remarkably long period of viral shedding and repeat hospitalization were caused in part by immunosuppression due to her MS treatment. Other contributing factors may include long-term corticosteroid use and complications of an invasive procedure (intrapulmonary hemorrhage). Our case contributes to the evidence in the literature that treatment with anti-CD20 monoclonal antibodies can result in persistent SARS-CoV-2 infection and the development of *de novo* mutations.

## Figures and Tables

**Table 1.** Basic metabolic panel and complete blood count at admission on patient’s three visits.

	November 2020	December 2020	March 2021
**Basic metabolic panel**			
Sodium (mmol/L)	135	136	140
Potassium (mmol/L)	3.3*	3.8	4.3
Chloride (mmol/L)	102	99	102
Carbon dioxide (mmol/L)	20*	29	23
Glucose (mg/dL)	113†	106	153†
Blood urea nitrogen (mg/dL)	9	13	11
Creatinine (mg/dL)	0.86	0.72	0.88
Calcium (mg/dL)	8.3*	8.2*	8.2*
**Complete blood count**			
White blood cell count (×10^3^ cells/uL)	4	8.1	16.2†
Neutrophil (%)	78.3†	69	75.9†
Lymphocyte (%)	12*	23.1	20.4
Monocyte (%)	9.1	6	3.2
Hemoglobin (g/dL)	13.5	12.2	11.6*
Platelet count (×10^3^ cells/uL)	344	178	361

* Below reference range.

† Above reference range.

**Table 2.** Inflammatory markers at admission on patient’s three visits.

	November 2020	December 2020	March 2021
Erythrocyte sedimentation rate (mm/h)	38	110	69
Procalcitonin (ng/mL)	0.12	0.84	2.37
Interleukin-6 (pg/mL)	48.52	-	74.77
Lactate dehydrogenase (U/L)	629	423	378
Ferritin (ng/mL)	3,374	4,654	3,574

## Data Availability

De-identified sequencing data from the patient’s SARS-CoV-2-positive samples will be made available on reasonable request.
